# Is ICU admission associated with chronic narcotic use? a 4-year follow up of ICU survivors

**DOI:** 10.1186/2197-425X-3-S1-A366

**Published:** 2015-10-01

**Authors:** PB Yaffe, MB Butler, RS Green, T Witter

**Affiliations:** Dalhousie University, Halifax, Canada

## Introduction

Patient comfort is a priority in the Intensive Care Unit (ICU). Narcotics are used to ensure optimal comfort and to facilitate patient management, including mechanical ventilation and other interventions. Previous studies indicate that chronic pain is common for an extended period after ICU discharge [1]. However, little data is available on the use of narcotic medications before and after ICU admission. We sought to describe narcotic use in this population over a multi-year time period.

## Objectives

To describe narcotic use before and after ICU admission, and to identify factors associated with chronic narcotic use up to 4 years after ICU discharge.

## Methods

Retrospective review of adult (≥ 18 years) patients admitted to an ICU in Halifax, Canada between January 1, 2005 to December 31, 2008. The dataset was created by merging hospital databases with a provincial medication database. Data collected included age, gender, length of ICU/hospital stay, interventions and complications during admission. We defined “naïve”, “intermittent”, and “chronic” narcotic status by abstinence, use in < 70%, or >70% of days for a given time period, respectively. We assessed narcotic use at 3 months prior to ICU admission, at discharge, and annually for up to 4 years following ICU discharge.

Statistical methods used were Welch's t-test, Wilcoxon Rank-Sum test, Fisher's test, McNemar's test, and logistic regression.

## Results

We included 2595 patients (mean age 46 yrs, 60.4% male). Reason for ICU admission was surgical in 48.6%, medical in 38.4% and undetermined in 13%. The population included both elective and emergent admissions.

In the 3 months prior to ICU admission, 76.9% were narcotic-naïve while 16.9% used narcotics intermittently, and 6.2% chronically. We found an increase in patients in the naïve category from 87.8% in the early post-ICU period to 95.6% at 48-month follow-up with a corresponding decreasing trend in intermittent (8.6% to 2.6%) and chronic (3.6% to 1.8%) narcotic usage, respectively.

On logistic regression, prolonged hospital length of stay was associated with chronic narcotic use, although this effect varied with time. Naïve baseline narcotic use was associated with less chronic narcotic use, and intermittent baseline use was not associated with chronic use.

## Conclusions

In our study, admission to an ICU was not associated with chronic narcotic use. Further research is required to confirm our findings in other health care environments.

## Grant Acknowledgment

Support from Capital District Health Authority operating grant
